# 627. Bioinformatic Approach to Design a *Plasmodium falciparum* PfRipr Multi-Epitope Vaccine Construct

**DOI:** 10.1093/ofid/ofad500.693

**Published:** 2023-11-27

**Authors:** Alexander J Laurenson, Emily Stucke, Ryan Scalsky, Matthew B Laurens

**Affiliations:** University of Maryland School of Medicine, Baltimore, Maryland; University of Maryland School of Medicine, Baltimore, Maryland; University of Maryland, School of Medicine, Baltimore, Maryland; Center for Vaccine Development and Global Health, University of Maryland School of Medicine, Baltimore, Maryland

## Abstract

**Background:**

Malaria infections remain an enormous contributor to global fatalities with an estimated death toll of 619,000 in 2021 and 77% of deaths being children aged under 5 years. Transmitted via mosquito, *Plasmodium falciparum* is the most lethal parasite of its genus but has evaded many treatment and vaccine efforts due to its complex life cycle and redundant invasion mechanisms. Epitope-based vaccines hold significant promise for malaria vaccine development due to their ease of development and ability to target dominant regions in antigenically variable pathogens. The recently characterized protein *P. falciparum* merozoite Rh5 interacting protein (PfRipr) is nonredundant, highly conserved, and essential for erythrocyte invasion, making it an ideal target for a bloodstage malaria vaccine.

Merozoite Invasion

**Figure:**
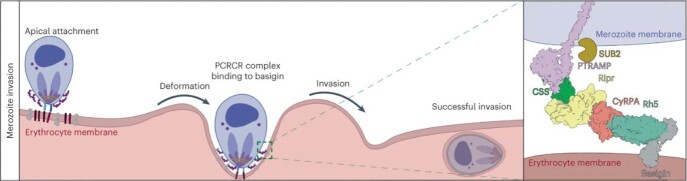

Model of binding and insertion of PRCR complex to erythrocyte basigin in merozoite invasion. Adapted from Scally et al. 2022.

**Methods:**

Using *P. falciparum* sequences collected from Burkina Faso and Uganda, we assessed the immunogenic potential of PfRipr epitopes with regard to T-cell receptor binding and B-cell recognition. T-cell receptor binding was predicted using NetMHCpan searching against MHC I and II alleles with high regional frequencies. Using an in-silico 3D model of PfRipr predicted via AlphaFold, tertiary structures of all PfRipr sample sequences were predicted via SWISS-MODEL then analyzed by ElliPro to identify linear and discontinuous B-cell epitopes. Putative epitopes were filtered using allele coverage, conservation, antigenicity, and allergenicity.

B-Cell Epitope Prediction

**Figure:**
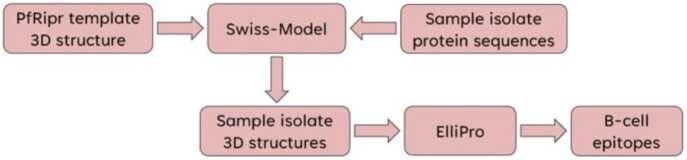

Protocol for B-cell epitope prediction using AlphaFold for template modeling, Swiss-Model for homology-based modeling of sample sequences, and ElliPro for continuous and discontinuous epitope prediction.​

Epitope Sorting and Filtering

**Figure:**
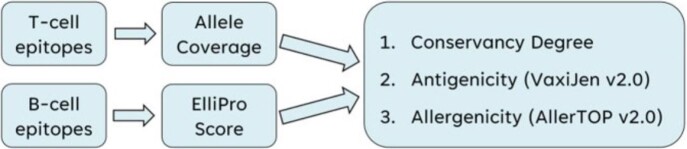

Protocol for sorting T-cell epitopes using Average Allele Coverage (percentage of alleles in which the protein sequence was identified as an epitope) and B-cell epitopes using ElliPro Score (Protrusion Index representing surface accessibility of the epitope).​

**Results:**

Between the two datasets, there were 19 matching epitopes with 7 MHC I, 9 MHC II, 1 linear, and 2 discontinuous. These epitopes were used to design a multi-epitope-based bloodstage vaccine construct against *P. falciparum*.

MHC Epitopes

**Figure:**
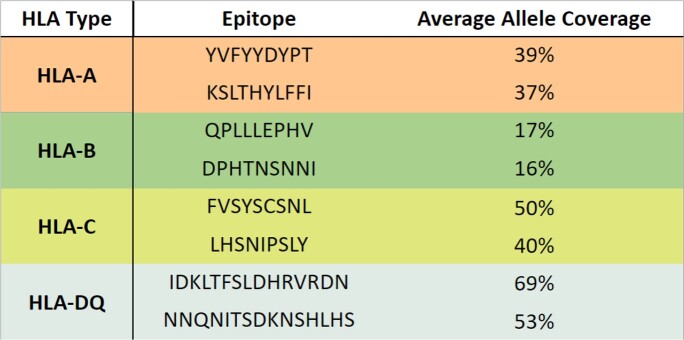

Partial list of MHC epitopes found in Burkina Faso and Uganda datasets.

B-Cell Epitopes

**Figure:**
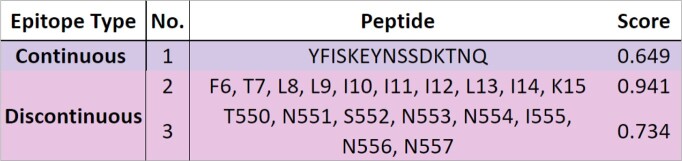

B-cell epitopes found in Burkina Faso and Uganda datasets.

PfRipr 3D Structure with B-cell Epitopes

**Figure:**
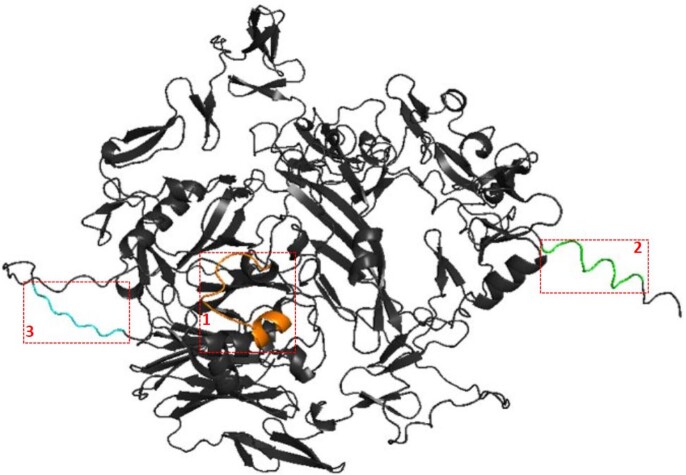

3D model of PfRipr predicted via AlphaFold with labeled B-cell epitopes

**Conclusion:**

To validate their predicted immunogenicity, epitopes can be further investigated using *in silico* protein stabilization and docking simulations, *in vitro* methods such as HLA stabilization or T-cell activation assays, and *in vivo* methods using transgenic mouse models. The pipeline of immunoinformatic analyses formulated in this project can be further applied to *P. falciparum* sequence datasets collected from other malaria endemic regions to develop vaccines effective against circulating strains.

Multi-Epitope Vaccine Construct

**Figure:**
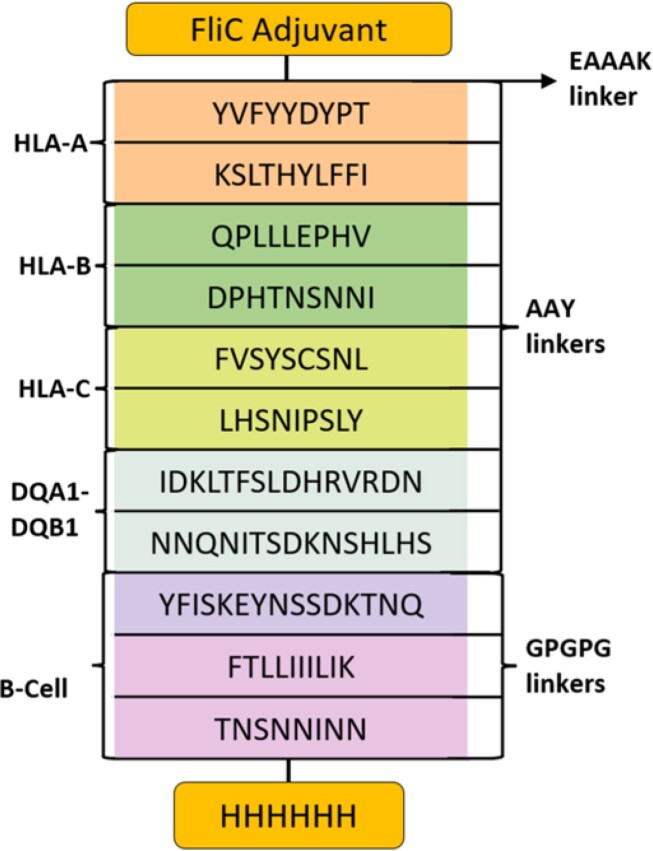

Schematic representation of hypothetical multi-epitope vaccine construct. FliC is added to the N-terminus to stimulate and amplify immune response. The EAAAK linker is used to separate the bifunctional fusion protein domains. AAY linkers are used to amplify MHC I and II epitopes and prevent junctional epitope binding. GPGPG linkers between B-cell epitopes function to minimize junctional epitopes and retain conformational-dependent immunogenicity. A six amino acid Histidine (6H) chain was added to the C-terminus to aid during lab purification processes.

**Disclosures:**

**All Authors**: No reported disclosures

